# Chloroplast genome and nuclear loci data for 71 *Medicago* species

**DOI:** 10.1016/j.dib.2024.110540

**Published:** 2024-05-17

**Authors:** Filipe de Sousa, Yann J.K. Bertrand, Alexander Zizka, Patrik Cangrén, Bengt Oxelman, Bernard E. Pfeil

**Affiliations:** a cE3c-Centre for Ecology, Evolution and Environmental Changes & CHANGE-Global Change and Sustainability Institute, Faculdade de Ciências, Universidade de Lisboa, 1749-016 Lisbon, Portugal; b Institute of Botany, Academy of Sciences of the Czech Republic, CZ-252 43 Průhonice, Czech Republic; c Department of Biology, Philipps-University Marburg, Karl-von-Frisch-Strape 8, 35043 Marburg, Germany; d Department of Biological and Environmental Sciences, University of Gothenburg, Medicinaregatan 7B, Göteborg 413 90, Sweden

**Keywords:** Sequence-capture, Phylogenetics, Genomics, Fabaceae

## Abstract

We present a dataset containing nuclear and chloroplast sequences for 71 species in genus *Medicago* (Fabaceae), as well as for 8 species in genera *Melilotus* and *Trigonella*. Sequence data for a total of 130 samples was obtained with high-throughput sequencing of enriched genomic DNA libraries targeting 61 single-copy nuclear genes from across the *Medicago truncatula* genome. Chloroplast sequence reads were also generated, allowing for the recovery of chloroplast genome sequences for all 130 samples. A fully-resolved phylogenetic tree was inferred from the chloroplast dataset using maximum-likelihoood methods. More than 80% of accepted *Medicago* species are represented in this dataset, including three subspecies of *Medicago sativa* (alfalfa). These data can be further utilised for phylogenetic analyses in *Medicago* and related genera, but also for probe and primer design and plant breeding studies.

Specifications Table

**Table:** 

Subject:	Biological Sciences
Specific subject area:	Phylogeny and Evolution
Type of data:	Processed sequence data, tables
Data collection:	The Illumina MiSeq high-throughput platform (San Diego, California, USA) was used to sequence enriched genomic DNA libraries. The MYBaits hybrid-capture method (MYcroarray, Ann Arbor, Michigan) was used for library enrichment targeting 61 single-copy nuclear loci. Probes for target enrichment were obtained from the *Medicago truncatula* genome (http://medicagohapmap.org). Sequence reads were processed using the CLC Assembly Cell software (CLC Bio, Aarhus, Denmark).
Data source location:	Department of Biological and Environmental Sciences, University of Gothenburg, Sweden
Data accessibility:	Repository name: Mendeley Data Data identification number: https://doi.org/10.17632/r5zzxg4xsw.1 Direct URL to data: https://data.mendeley.com/datasets/r5zzxg4xsw/1

## 1 Value of the Data

• These data correspond to the first genomic dataset generated by high-throughput sequencing that includes the vast majority of *Medicago* species, including species never before sampled in molecular phylogenetic studies.
• Other researchers can use these data directly for phylogenetics and population studies in *Medicago* and related genera, but also for probe and primer design.
• Chloroplast genome data contributes to the understanding of phylogenetic relationships among *Medicago* species and can be used in future comparative studies.

## 2 Background

The dataset presented herein was generated in the context of a research project on the phylogeny of genus *Medicago* (Fabaceae), aimed at exploring biological causes of phylogenetic incongruence affecting tree inference in this genus, namely incomplete lineage sorting, paralogy and hybridisation [1]. The availability of an annotated genome for genus *Medicago* enabled the development of a probe set to obtain sequence data using innovative sequence-capture techniques and high-throughput sequencing. Sequence capture targeted the 61 nuclear gene set, but chloroplast genomes were also sequenced in the process, allowing for the compilation of the chloroplast whole-genome dataset. Part of the sequence data generated in the context of this research have supported several different publications [2,3,4,5]. However, all sequences in the present dataset have been newly generated from the original raw reads using a pipeline that automated allele phasing and SNP calling. Sampling includes 71 species and subspecies in genus *Medicago* L. [6], as well as two species in genus *Trigonella* L. and six species in genus *Melilotus* Mill., in a total of 130 samples. Genera *Trigonella* and *Melilotus* form the sister-group to genus *Medicago* and were sampled to be used as outgroups in phylogenetic inference.

## 3 Data Description

The data set presented herein is divided into two folders, one for nuclear data and another for chloroplast data, along with two tables describing the data and sampling.

Nuclear data is organised into 61 unaligned multifasta files corresponding to all targeted genes. Each multifasta file contains one consensus sequence and two phased sequences (allele 0 and 1) for each sample present in the file. Samples for which only the consensus sequence is available are assumed to be homozygous in that locus. Genes were sampled from 20 genomic blocks of three to four genes, distributed in eight chromosomes, each chromosome containing two or three unlinked genomic blocks. Genes were chosen according to the following criteria: minimum distance between each gene within a genomic block = 30Kbp; minimum gene length = 2Kbp; maximum intron length=500bp. All genes were in single-copy in the Medicago genome and had homologues in other plant genomes [2]. Gene names and references, their location in each chromosome of the *Medicago* genome, the corresponding genomic block and the number of sequences present in each multifasta file are presented in “Table A – Nuclear Data”.

Chloroplast data is organised into 130 fasta files, one for each sample. Sample names, their corresponding genus, species, accession, number of base pairs and percentage coverage in chloroplast sequences, as well as accession numbers in the European Nucleotide Archive, where raw sequence data was stored (https://www.ebi.ac.uk/ena) are presented in the “Table B– Samples”. The minimum coverage per sample in the chloroplast genome dataset is 40%, and the maximum coverage is 99.6%, with a median of 92% for the 130 samples.

Table 1 shows the completeness of the nuclear and chloroplast datasets, for each of the three plant genera sampled and an averaged total, based on the comparison between reference sequence lengths and the number of sequenced base pairs (for the 61 gene dataset, estimates were made from consensus sequences).

**Table 1:** Completeness of the nuclear and chloroplast datasets.

genus	N species	N individuals	Coverage 61 loci dataset	Coverage chloroplast dataset
*Medicago*	71	122	85,1%	88,4%
*Trigonella*	2	2	63,1%	68,2%
*Melilotus*	6	6	82,1%	92,0%
Total	79	130	84,6%	88,2%

## 4 Experimental Design, Materials and Methods

Sampling, DNA extraction, target enrichment and sequencing were done as described in [2]. In brief, genomic DNA libraries were enriched for a target of 61 nuclear loci using the MYBaits hybrid-capture method (MYcroarray, Ann Arbor, Michigan) and sequenced with Illumina MiSeq (San Diego, California, USA) at the Genomics Core Facility of the University of Gothenburg, Sweden.

Read trimming, quality filtering and mapping were performed using the CLC Assembly Cell software (CLC Bio, Aarhus, Denmark). Raw paired-end sequence reads were trimmed of adapter sequence and filtered for quality with a minimum quality score of 20. A first round of mapping was done against the 61 nuclear gene reference sequences used to design probes for target enrichment (sequences obtained from the *Medicago truncatula* genome). As chloroplast DNA present in the enriched genomic libraries was also sequenced, reads were also mapped against a whole-chloroplast reference sequence of *Medicago truncatula*, retrieved from GenBank (accession AC093544). Mapped reads were converted into sequences with the program samtools [7], using the mpileup tool. Consensus sequences generated for each sample and each gene, containing indels not present in the original reference, were used as reference for a second round of mapping, followed by phasing of two alleles using samtools phase. Allele and consensus sequences from the second round of mapping were generated using mpileup without the reference sequence option, to avoid erroneous base calling where read depth was low [8].

**Fig. 1:**
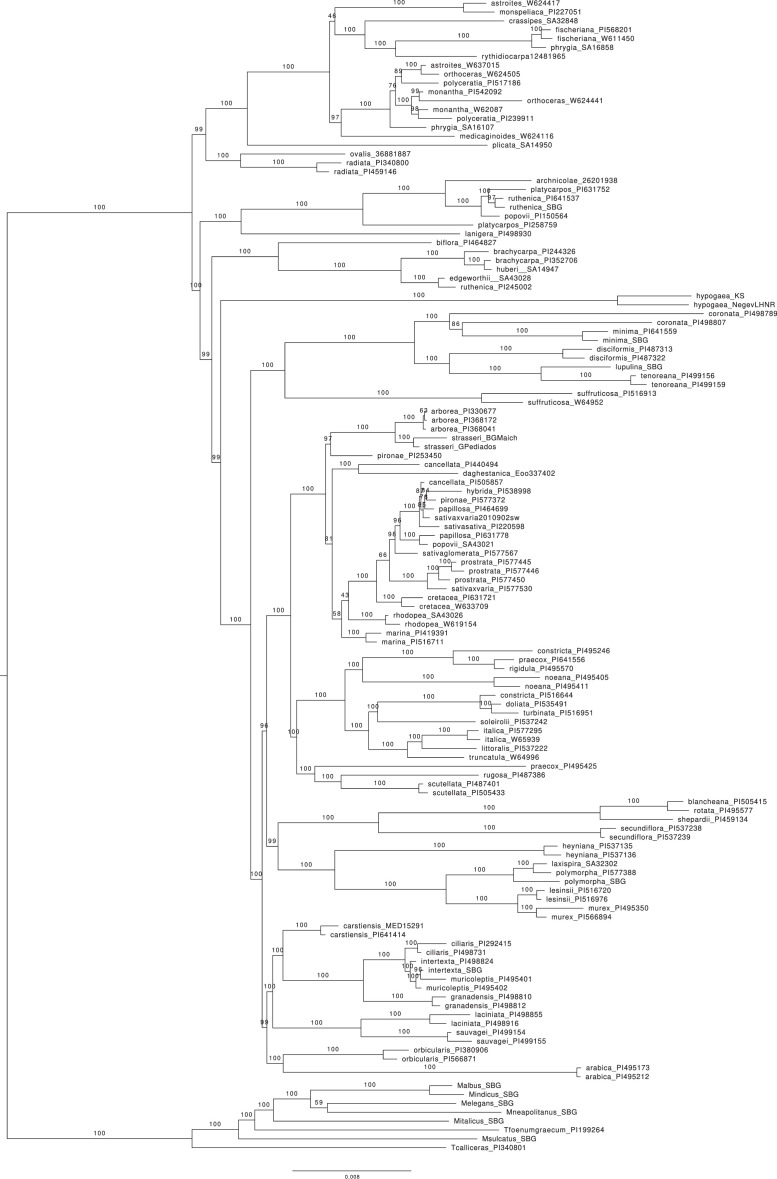
Phylogenetic tree Phylogram obtained from the maximum-likelihood analysis of the chloroplast data on IQTree. Branch support values were obtained from 1000 ultrafast bootstrap replicates.

Chloroplast genome sequences used to infer a phylogenetic tree using maximum-likelihood methods (Fig. 1). Whole-chloroplast sequence files of 130 samples were aligned using MAFFT v. 7.3 [9]. Sites containing gaps in more than 50% of sequences were deleted from the alignment using TrimAl v. 1.2 [10]. A maximum-likelihood analysis of the alignment was run using IQTree v. 2.2.2.3 [11], under the GTR substitution model and a discrete four category gamma model of site rate heterogeneity, as determined by model testing in IQTree and the corrected Akaike Information Criterion. Support for the best tree was obtained with 1000 ultrafast bootstrap replicates. The analysis ran on the CIPRES Science gateway [12]. The analysis recovers a fully resolved tree with high bootstrap support for most nodes, including those corresponding to samples with lower coverage, thus confirming the utility of the chloroplast dataset for phylogenetic analyses. The main clades recovered can be identified in earlier tree inferences [1], although previously used markers did not recover supported relationships among clades.

## Limitations

None.

## Ethics Statement

The authors have read and follow the ethical requirements for publication in Data in Brief and confirm that the current work does not involve human subjects, animal experiments or any data collected from social media platforms.

## CRediT authorship contribution statement

**Filipe de Sousa:** Conceptualization, Data curation, Validation, Writing – original draft. **Yann J.K. Bertrand:** Software, Validation, Writing – review & editing. **Alexander Zizka:** Data curation, Validation. **Patrik Cangrén:** Data curation, Validation. **Bengt Oxelman:** Methodology, Supervision. **Bernard E. Pfeil:** Conceptualization, Methodology, Supervision, Writing – review & editing.

## Data Availability

Nuclear and chloroplast sequence data for 130 samples from genera Medicago, Trigonella and Melilotus (Fabaceae) (Original data) (Mendeley Data).
